# Supplementary material for: Understanding coverage of antenatal care in Palestine: Cross-sectional analysis of Palestinian Multiple Indicator Cluster Survey, 2019–2020
Source: PLoS One. 2024 Feb 2;19(2):e0297956. doi: 10.1371/journal.pone.0297956 (PMC10836664; doi:10.1371/journal.pone.0297956)
Supplement: Attachment — Submitted filename: Response to reviewers 2023 Aug 19.docx [file pone.0297956.s001.docx]

Cover letter

Date: August 20, 2023

From: Masako Horino

Re: Submission ID: PONE-D-23-16337: “Understanding coverage of antenatal care in Palestine: Cross-sectional Analysis of Palestinian Multiple Indicator Cluster Survey, 2019-2020”

We are grateful to the editors and reviewers for their time and constructive comments on our manuscript. Please, find enclosed a revised manuscript addressing your comments and suggestions. We are enclosing two copies: one unmarked revised version and one where the most significant changes (mostly in response to your suggestions) have been highlighted. Below we provided point-by-point response to each of your and the reviewers’ comments.

**Editor’s Comments:**

**1. I want you to incorporate a brief literature review on the topic to show what previous studies have been done on antenatal care in Palestine, or the determinants in antenatal care in general. This will strengthen the Introduction and Significance section of the paper as well as provides justification for the choice of variables that you will be including in your analysis. In connection to this, please provide justification for the variables that you have included in your analysis (i.e., why did you include these variables in your analysis? Which previous studies justify these choices?)**

Thank you for your comments. The following paragraph was included in the introduction section.

“Previous studies have reported several factors associated with adequate antenatal care: initiation of care in the first trimester of pregnancy, residence in an urban area, especially close to an ANC facility, secondary or higher education, small household size (fewer than five members), higher socioeconomic status, lower parity (fewer than four live births), having health insurance, and being married vs single [3,7-10]. Furthermore, early pregnancy registration, clinical competence of the healthcare provider, and attentive interactions are also thought to be significant predictors of achieving the WHO recommended number of eight ANC contacts in pregnancy [7,10]. Globally, the percentage of women who initiate ANC in the first trimester has increased from 41% in 1999 to 59% in 2013. However, there is still a significant disparity in the receipt of any ANC coverage between low-income countries (24%) and high-income countries (82%) [5].”

**2. I appreciate the candid description of their methodology. However, I would like you to discuss your: (1) covariate selection strategy (i.e., how did you pick the variables to include in your regression model), and (2) any testing for overdispersion considering that you have used a Poisson model and the results of such testing. I am also of the opinion that one of the reason why your initial model failed to converge is that you are not supposed to use MLE methods for survey data.**

Thank you for your comments. For the first comment , we included the following sentence to the methodology section: “Then, a design-based multivariable log-binomial regression model was fitted to adjust for selected covariates that were identified to be the determinants of ANC coverage, based on the previous literature, including women’s age, education level, number of parities, wealth index, area of residence, type of ANC provider, and timing of first antenatal contact [3,7,9,10,25].”

For the second comment, we fitted a design-based robust Poisson model to relax the assumption of mean equals variance, and the estimated variances are larger than the conventional Poisson models under the simple random sampling assumption.

**3. Why not use Poisson regression for the univariate analysis as well? This is to ensure that crude and adjusted models are actually comparable.**

We have checked analysis and the Poisson regression was used for the univariate analysis as suggested. The footnote on Table 3 was edited accordingly.

**4. Consider testing for departure from linearity assumption for quantitative variables like parity to reduce parameters estimated.**

Thank you for your comment. As suggested, we tested for nonlinearity of age and parity by including both the continuous variables and quadratic terms in the design-based Poisson regression model. Since the quadratic term of age did not reach statistically significant level (p<0.05) suggesting that the relationship of age is linear, age was fitted as a continuous variable. However, both the quadratic term of parity and parity as a continuous variable were significantly associated with ANC coverage (p<0.05).

The following footnote was added to Table 3 to denote that a quadratic term of parity was included in the model: “c Quadratic term of parity was included in design-based robust multivariable Poisson regression to calculate the adjusted PrR of inadequate contact per unit increase in number of live births.”

**5. I found the Discussion section to be a little bit lacking especially on what you could recommend to address the issues that you have found. Please strengthen it by putting recommendations to address the issues that you have identified.**

Thank you for your comments. The following recommendation was added to the discission section.

“To ensure that women in Palestine are adequately covered with ANC services, health facilities providing antenatal care in Palestine should update their guidelines per the updated WHO recommendation for the number of antenatal contacts and increase awareness of pregnant women on the importance of antenatal care in reducing the risk of adverse birth outcomes and pregnancy complications. Health facilities may consider using Short Message Service Alerts (SMSs) to ensure adherence to the WHO guidelines for the number of ANC visits [37]. Furthermore, continuous evaluation of ANC provision will benefit the Palestinian health systems by helping to prioritize the appropriate strategies, to meet the needs of Palestinian pregnant women, and to improve the quality of ANC services [16].”

**6. There are minor grammatical errors. Please have the manuscript reviewed by an English editor or a native speaker before resubmitting so that we can go straight to publication.**

Thank you for your comments. The authors have reviewed the English grammar carefully and made edits accordingly.

**Reviewers' comments:**

**Reviewer #1: Well done analyses, particularly commend use of prevalence ratios. Interesting and of course disturbing findings, although I note that overall ANC is higher than for example most of Africa. It might have been nice to compare ANC rates to other countries in the middle-eastern region as a normative comparison.**

Thank you for your comment. We have included the following sentences in the discussion section.

“Although the health systems in Palestine are challenged by recurrent conflicts, movement restrictions, insufficient resources and funding, shortage of health care providers, and complex politics, ANC coverage in Palestine is comparable to that in neighboring countries in the Middle Eastern Region [17,21]. Based on the recent national household surveys in both Jordan and Egypt, 67.8% and 60.6% of women, respectively, initiated ANC contact in the first trimester and completed at least eight ANC visits [28].”

**Reviewer #2: Is the manuscript technically sound, and do the data support the conclusions?**

**Yes. The presentation of results and discussion followed the objectives of the study. Conclusions were directly supported by the results of the study. Limitations such as recall bias were also explicitly stated in the Discussion and Conclusions.**

**Is the manuscript presented in an intelligible fashion and written in standard English?**

Yes. With some minor grammatical errors that need to be corrected prior to publication.

**Line 39: Results: Overall, 28% of women did not *meet* the WHO recommendation of eight or more**

Corrected.

**Line 112: The *deficiency or lack of information* needed for management of ANC**

Corrected.

**Line 191: Table 1 summarizes the characteristics of 2,028 women aged between 15 to 49**

***However, Table 1 in Line 208 indicates n= 2,191. Kindly reconcile the values.**

Corrected.

We hope that the above responses and the enclosed revised version of the manuscript properly address the concerns of the reviewers; but please, let us know if you have any additional questions or concerns.

Sincerely,

Masako Horino, representing all coauthors
