# Supplementary material for: Understanding coverage of antenatal care in Palestine: Cross-sectional analysis of Palestinian Multiple Indicator Cluster Survey, 2019–2020
Source: PLoS One. 2024 Feb 2;19(2):e0297956. doi: 10.1371/journal.pone.0297956 (PMC10836664; doi:10.1371/journal.pone.0297956)
Supplement: Attachment — Submitted filename: Response to reviewers 2023 Dec 8.docx [file pone.0297956.s002.docx]

Date: December 9, 2023

From: Masako Horino

Re: Submission ID: PONE-D-23-16337: “Understanding coverage of antenatal care in Palestine: Cross-sectional Analysis of Palestinian Multiple Indicator Cluster Survey, 2019-2020”

We would like to thank the editors and reviewers for their time and constructive comments on our manuscript. Please, find enclosed a revised manuscript addressing your comments and suggestions. We are enclosing two copies: one unmarked revised version and one where the most significant changes (mostly in response to your suggestions) have been highlighted. Below we provided point-by-point response to each of your and the reviewers’ comments.

**Additional Editor’s Comments:**

**Thanks for working on this. May I clarify why variables in Table 1 do not add up to 2028?**

Thank you for your comment. The variables in Table 1 did not add up to 2028, because the analysis was weighted by the original (non-normalized) weight. However, we have now applied normalized weights to match the analytical total sample size to the observed total sample size, which we hope will avoid confusion in the sample size.

**Also please add brief commentary on the current issue in Palestine. Consider the comments of Reviewer 2 as well.**

Thank you for your comment. The following sentence has been included in the discussion section (lines 332-334).

“However, the conflict that began on the 7th of October 2023 has severely destroyed health system infrastructure in the Gaza Strip and limited the movement of civilians in the West Bank, which have likely impeded access to and coverage of ANC in Palestine.”

**Reviewer #2s' comments:**

**1) health facilities should update their guidelines per the updated WHO recommendations on the number of ANC contacts: There is no explicit mention in the study about the existing local policies on antenatal care services which should be the basis of the recommendation to update the local guidelines.**

Thank you for your comment. We have decided not to include this recommendation.

**2) health facilities may consider using SMS to ensure adherence to WHO guidelines: There is no explicit mention in the study about women's access to telecommunication services, nor the acceptability and availability of these services for the women and their families. This recommendation should also consider the capacity of the health facilities to provide SMS services. Recommendations should be supported by the findings of the study.**

Thank you for your comment. We have decided not to include this recommendation.

**3) continuous evaluation of ANC provision: The study mentioned in its introduction that there is "little reported national data" and "lack of information needed" which can be presumed to be related to a lack of accessible administrative reports on the provision of health services including ANC at the national scale. As such, it seems that there is a need to establish or strengthen the monitoring and evaluation mechanism for the provision of ANC services instead of "continuously evaluating" its provision.**

Thank you for your comment. The following sentence has been added to the discussion section (lines 340-343).

“Practical approaches to continuously provide ANC services and strengthening its monitoring and evaluation mechanism in Palestine should be further considered.”

We hope that the above responses and the enclosed revised version of the manuscript properly address the concerns of the reviewers; but please, let us know if you have any additional questions or concerns.

Sincerely,

Masako Horino, representing all coauthors
